# What is the diagnostic accuracy of single nerve conduction studies and muscle ultrasound to identify critical illness polyneuromyopathy: a prospective cohort study

**DOI:** 10.1186/s13054-018-2281-9

**Published:** 2018-12-17

**Authors:** Daniel A. Kelmenson, Dianna Quan, Marc Moss

**Affiliations:** 1 Division of Pulmonary Sciences & Critical Care Medicine, University of Colorado School of Medicine, RM 9023, Mail Stop C272, 12700 East 19th Avenue, Aurora, CO 80045 USA; 2 Department of Neurology, University of Colorado School of Medicine, Aurora, CO USA

**Keywords:** Critical care, Critical care outcomes, Critical illness, Muscle weakness, Muscular diseases, Polyneuropathies

## Abstract

**Background:**

Critical illness polyneuromyopathy (CIPNM) is a major cause of weakness in intensive care unit (ICU) patients, but current diagnostic tests are limited. We evaluated the generalizability and validity of single nerve conduction studies (NCS) and muscle ultrasound testing to identify CIPNM, and we also assessed the ability of muscle ultrasound to prognosticate patient outcomes.

**Methods:**

This was a prospective cohort study of mechanically ventilated medical, cardiac, surgical, and neurosurgical ICU patients. We performed weekly strength testing, NCS, electromyography (EMG), and muscle ultrasound. We calculated the sensitivity, specificity, and other test characteristics of single NCS and muscle ultrasound, and we used multivariable regression models to assess the prognostic ability of muscle ultrasound.

**Results:**

Ninety-five patients were enrolled. The incidence of probable CIPNM was 18% and did not differ significantly by type of ICU (*p* = 0.49). For diagnosing probable CIPNM, the peroneal motor NCS had a sensitivity of 94% (95% confidence interval (CI) 71–100%) and specificity of 91% (95% CI 82–96%), the sural sensory NCS had a sensitivity of 100% (95% CI 80–100%) and specificity of 42% (95% CI 31–54%), and abnormal muscle ultrasound echogenicity had a sensitivity of 82% (95% CI 48–98%) and specificity of 57% (95% CI 43–70%). Abnormal echogenicity was associated with reduced likelihood of discharge to home (9% vs 50%, *p* = 0.0001), fewer ICU-free days (median 3 (interquartile range 0–15) days vs 16 (9.3–19.3) days, *p* = 0.0002), and increased ICU mortality (42% vs 12%, *p* = 0.004).

**Conclusions:**

In a diverse cohort of critically ill patients, single NCS and muscle ultrasound achieved diagnostic accuracy for patients at risk for CIPNM. The routine utilization of these tests could be beneficial for all critically ill patients at risk for CIPNM.

**Electronic supplementary material:**

The online version of this article (10.1186/s13054-018-2281-9) contains supplementary material, which is available to authorized users.

## Background

Each year, approximately 1 million critically ill mechanically ventilated patients worldwide develop intensive care unit-acquired weakness (ICUAW) [1]. The prevalence of ICUAW varies widely depending on factors such as the presence of sepsis and multi-organ failure [2–14]. Weakness may be related to deconditioning (weakness without electrophysiologic abnormalities) or critical illness polyneuromyopathy (CIPNM; weakness with electrophysiologic abnormalities) [6, 9, 10, 15, 16]. Differentiating deconditioning from CIPNM is clinically important, as these two groups of patients have different outcomes and distinct courses of recovery [17].

The diagnosis of CIPNM can be challenging. Muscle strength testing is difficult to perform in acutely ill patients and does not differentiate CIPNM from deconditioning [18]. Although nerve conduction studies (NCS) and needle electromyography (EMG) can delineate CIPNM from deconditioning, these tests are time-consuming, mildly invasive (for EMG), use expensive equipment, and require specialized training.

A number of simplified screening tests for CIPNM have been proposed. NCS of a single motor nerve (such as the peroneal, also known as fibular, nerve) or sensory nerve (such as the sural nerve) may be a relatively accurate screening test for CIPNM [11–13]. The advantages of single NCS include shorter testing duration (5–10 min vs 60–90 min for full NCS/EMG), noninvasiveness, and no need for volitional patient movement. Electrophysiologic abnormalities are associated with deleterious outcomes in critically ill patients, even in the absence of weakness [17, 19]. However, a prior study examining the accuracy of single peroneal and sural NCS as screening tests for CIPNM focused almost exclusively on patients with severe sepsis [11]. Therefore, the generalizability of the accuracy of single NCS in screening for CIPNM is relatively unknown. Muscle ultrasound is also a promising technique to diagnose weakness by examining decreases in muscle thickness or changes in appearance (increased echogenicity) [20–34]. Muscle ultrasound has the potential advantage of being a relatively quick and noninvasive test that utilizes equipment present in most intensive care units (ICUs). However, the accuracy of ultrasound changes in muscle thickness or echogenicity to diagnose CIPNM remains unclear, and it is unknown if ultrasound provides prognostic information beyond that obtained from NCS/EMG.

The main purposes of our study were to determine the generalizability of unilateral peroneal and sural NCS for screening for CIPNM in a broader population, including critically ill cardiac, surgical, and neurosurgical patients, and to evaluate the accuracy of muscle ultrasound in screening for CIPNM and prognosticating outcomes in critically ill patients.

## Methods

This was a prospective observational cohort study conducted at the University of Colorado Hospital, a tertiary academic institution. We enrolled patients from the medical, cardiac, surgical, and neurosurgical ICUs. The study was approved by the Colorado Multiple Institutional Review Board. All subjects or their proxies provided written informed consent prior to inclusion in the study. If a proxy was used for the initial consent, re-consent of the subject was attempted during the hospital course. Some of these data was previously presented as an abstract at the American Thoracic Society International Conference 2018.

We identified potentially eligible patients using daily screening for mechanically ventilated patients in the electronic health record of our hospital. For medical, cardiac, and surgical ICU patients, the study inclusion criteria were: 1) intubation for > 48 h with hypoxemia or hypercarbia in conjunction with severe sepsis or septic shock; or 2) ICU stay for > 48 h with multi-organ dysfunction and acute respiratory failure (PaO_2_/FiO_2_ < 250) requiring mechanical ventilation. For neurosurgical ICU patients, the inclusion criterion was intubation for > 48 h with nontraumatic subarachnoid or intracerebral hemorrhage. Exclusion criteria for all ICUs included age < 18 years, pre-existing neuropathy or myopathy, pharmacologic paralysis, pregnancy, being a prisoner, time on mechanical ventilation and ICU stay of > 7 days, inability to perform NCS/EMG on at least one arm and one leg (e.g., due to amputation or overlying equipment), or patient/physician refusal to participate in the study.

After enrollment, we collected baseline information on demographics, comorbidities, and Sequential Organ Failure Assessment (SOFA) score. Each week, patients underwent Glasgow Coma Scale (GCS) scoring, and muscle strength testing was attempted using Medical Research Council (MRC) scoring of six bilateral muscle groups with a maximum score of 60. ICUAW was defined as an MRC score of less than 48 [6].

Weekly NCS/EMG testing was performed with a Natus Neurology Nicolet Viking EDX (Middleton, WI, USA) according to previously described standard procedures [11, 35]. Repetitive stimulation of the median motor nerve was performed to exclude neuromuscular junction defects, and F-waves were recorded from the tibial nerves to screen for proximal nerve root disease (e.g., Guillain-Barre Syndrome). The bilateral sural, radial, and median sensory NCS were recorded using standard procedures [11]. The bilateral peroneal, tibial, and median motor NCS were recorded using surface electrodes over the extensor digitorum brevis, abductor hallucis brevis, and abductor pollicis brevis muscles, respectively. The compound motor action potential (CMAP) responses were elicited from standard distal and proximal sites of stimulation to calculate a conduction velocity and to assess for the presence of conduction block or temporal dispersion. After reviewing the studies and excluding patients with defects in neuromuscular transmission or primary/acquired demyelination, the sensory nerve action potential (SNAP) and CMAP amplitudes were analyzed for abnormalities. Unilateral concentric needle EMG examination was then performed on two upper extremity and two lower extremity muscles, one proximal and one distal in each limb, assessing insertional activity, spontaneous activity, activation, motor unit potential morphology, and recruitment pattern. NCS and EMG are prone to differences in interexaminer reliability but maintain high intraexaminer reliability [36]. Therefore, all NCS/EMG examinations were performed by one electrophysiology-trained physician, who was not blinded to the results of the index tests or reference standard.

For muscle ultrasound, we used a Philips Sparq machine (Amsterdam, Netherlands) with a linear-array transducer with standardized gain and varying depth based on the amount of overlying soft tissue and muscle size. The patients were examined in the supine position with extended limbs and relaxed muscles. We performed bilateral scans at standardized sites on the mid-biceps (halfway between the tip of the acromion and antecubital skin crease with forearm supinated), anterior mid-forearm (halfway between the antecubital skin crease and ulnar styloid with forearm supinated), and mid-thigh (halfway between the anterior superior iliac spine and superior midline border of the patella). We measured muscle thickness and echogenicity in the axial plane (perpendicular to the underlying bone or interosseous membrane) while avoiding compression of overlying soft tissues. To quantify muscle echogenicity, we utilized the visual four-point Heckmatt score that correlates with clinical and histologic neuromyopathy [29, 33]. All muscle ultrasounds were performed before NCS/EMG by one trained examiner to minimize issues of interexaminer reliability. Only medical, cardiac and surgical ICU patients underwent weekly muscle ultrasound due to machine availability. Weekly NCS/EMG/ultrasounds stopped once the patient left the ICU, died, developed CIPNM or completed four weekly examinations.

The primary outcomes for this study were the sensitivity and specificity of the unilateral peroneal motor and sural sensory nerves for diagnosing CIPNM, using the previously reported most accurate cutoff amplitudes to define test positivity for the peroneal and sural nerves (below 0.65 mV for peroneal and 4 μV for sural) and a reference standard electrophysiologic definition of CIPNM based on established criteria [11]. Patients were diagnosed with CIPNM if they had: 1) SNAP amplitudes less than 80% of the lower limit of normal in two or more nerves; and 2) CMAP amplitudes less than 80% of the lower limit of normal in two or more nerves without conduction block. NCS were categorized as normal or abnormal using standard normal values for the electrophysiology laboratory (normal amplitude > 1 mV for the peroneal nerve and > 10 μV for the sural nerve). We used this purely electrophysiologic definition of CIPNM (hereafter referred to as probable CIPNM) as our reference standard as we anticipated that most patients would not be awake and able to participate in voluntary MRC and EMG testing [8]. If the patient could participate in testing, MRC and EMG were used to classify the diagnosis definitively as neuropathy, myopathy, both, or neither. For patients diagnosed with probable CIPNM, the electrophysiological testing results at the time the diagnostic criteria were fulfilled were used in all analyses. For the remaining patients who did not meet the diagnostic criteria for probable CIPNM, data from their last electrophysiological tests were used in all analyses. Muscle ultrasound does not have established cutoffs to define abnormal changes in muscle thickness or echogenicity that are associated with CIPNM. We thus evaluated whether decreased muscle ultrasound thickness or increased echogenicity were accurate screening tests for probable CIPNM and if these muscle ultrasound abnormalities added prognostic information on patient outcomes to that obtained from NCS/EMG. We built a multivariable regression model with selected predictors of age (continuous variable), gender (binary variable), SOFA score for disease severity (continuous), CIPNM status (probable CIPNM vs none, binary), and muscle ultrasound echogenicity (abnormal vs normal, binary). The main predicted outcome for assessing the incremental prognostic information conveyed by muscle ultrasound was hospital discharge disposition (home vs not home) using a nominal logistic regression model with coefficient statistical significance assessed using a Wald test. We also examined the outcomes of ICU-free days and ICU mortality in secondary analyses.

We followed the Standards for Reporting Diagnostic Accuracy (STARD) 2015 guidelines for reporting diagnostic accuracy studies [37]. For our sample size calculation, since prior studies showed ~ 95% sensitivity of the peroneal motor and sural sensory nerves for CIPNM diagnosis [11–13], for a test with 95% sensitivity, two-sided 95% confidence interval (CI) width of 10%, and probable CIPNM prevalence of 20%, 92 patients would be needed [38]. Although our focus for these screening tests was sensitivity, these same prior studies showed ~ 75% specificity of the peroneal nerve for CIPNM diagnosis and for a test with 75% specificity, two-sided 95% CI width of 10%, and probable CIPNM prevalence of 20%, 91 patients would be needed [38]. Baseline data are presented as counts and percentage or medians and interquartile range (IQR). We used chi-square tests for categorical variables and *t* tests or Wilcoxon tests for continuous variables. Outcomes are presented as percentages for binary outcomes and medians and IQR for continuous outcomes. All analyses were performed using JMP Pro 13 (Buckinghamshire, England). A *p* value less than 0.05 was considered statistically significant and all significance tests were two-sided. There was no adjustment performed for multiple comparisons.

## Results

**Fig. 1:**
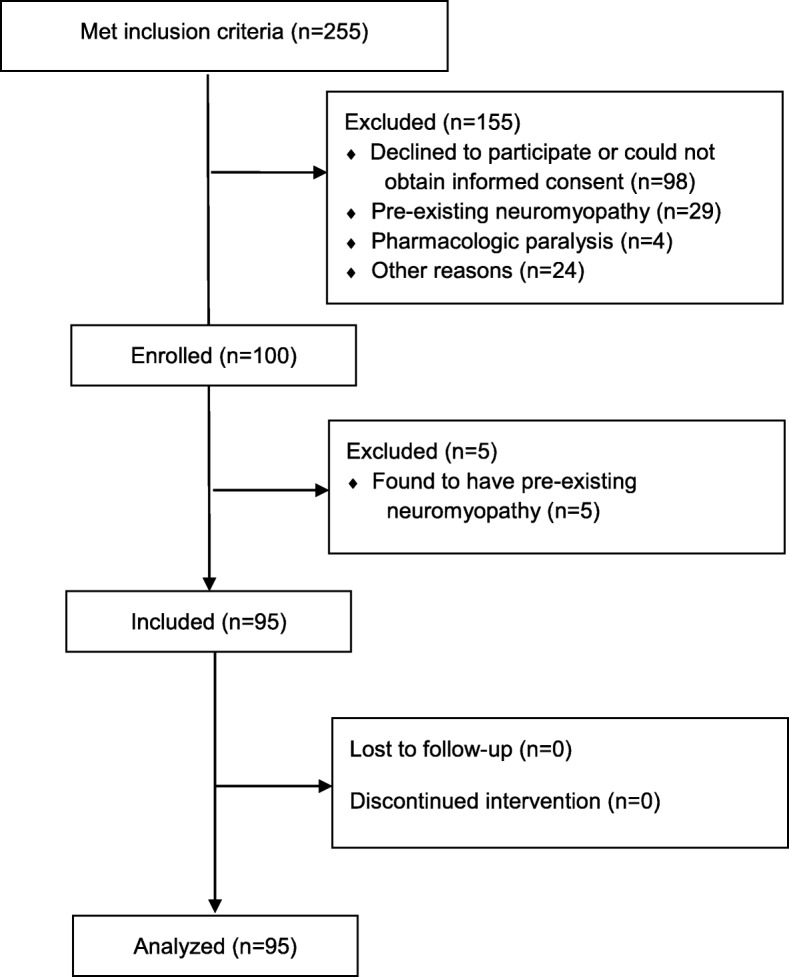
Study flow chart

From December 2015 to April 2018, 255 mechanically ventilated patients met inclusion criteria and 155 were excluded (Fig. 1), with the most common reasons including an inability to obtain informed consent or refusal to participate (*n* = 98, usually from lack of available proxy), pre-existing neuromyopathy (*n* = 29), and pharmacologic paralysis (*n* = 4). We initially enrolled 100 patients. After enrollment and initial NCS/EMG were performed, five subjects were discovered to have a history of pre-existing neuromyopathy so they were excluded from further analysis. Thus, the final cohort included 95 patients (Table 1). No patients withdrew from the study and outcomes were collected on all patients.

**Table 1:** Baseline demographic and clinical characteristics

Variables	Subjects (*n* = 95)	No CIPNM (*n* = 78)	Probable CIPNM (*n* = 17)
ICU location			
Medical	45 (47)	37 (47)	8 (47)
Cardiac	9 (9)	9 (12)	0 (0)
Surgical	13 (14)	10 (13)	3 (18)
Neurosurgical	28 (29)	22 (28)	6 (35)
Age, years	59 (43–70)	56 (43–65)	72 (59–77)
Gender, female	42 (44)	37 (47)	5 (29)
Race			
White	68 (72)	56 (72)	12 (71)
Black	13 (14)	12 (15)	1 (6)
American Indian or Alaska Native	3 (3)	2 (3)	1 (6)
Asian	1 (1)	1 (1)	0 (0)
Other or not reported	10 (11)	7 (9)	3 (18)
Ethnicity, Hispanic	13 (14)	10 (13)	3 (18)
Body mass index, kg/m^2^	28.7 (25–32.3)	28 (24.6–31.9)	29.4 (27.7–37.9)
Primary reason for admission			
Subarachnoid hemorrhage	19 (20)	14 (18)	5 (29)
Pneumonia	17 (18)	15 (19)	2 (12)
Encephalopathy	9 (9)	7 (9)	2 (12)
Intracerebral hemorrhage	8 (8)	7 (9)	1 (6)
Postoperative	7 (7)	7 (9)	0 (0)
Nonpulmonary sepsis	7 (7)	4 (5)	3 (18)
Gastrointestinal bleed	5 (5)	2 (3)	3 (18)
Congestive heart failure	3 (3)	3 (4)	0 (0)
ARDS	3 (3)	3 (4)	0 (0)
Myocardial infarction	2 (2)	2 (3)	0 (0)
Other	15 (16)	14 (18)	1 (6)
Hospital length of stay, hours	108 (66–157)	108 (66–152)	116 (76–162)
Time on mechanical ventilation, hours	83 (63–133)	83 (63–134)	84 (69–135)
Central nervous system disease	11 (12)	8 (10)	3 (18)
Alcohol use disorder	23 (24)	16 (21)	7 (41)
Diabetes	12 (13)	10 (13)	2 (12)
HIV	0 (0)	0 (0)	0 (0)
Total SOFA score	9 (6–12)	9 (6–11)	12 (8–14)
Total GCS score (eyes + motor)	4 (2–9)	7 (2–9)	2 (2–7)

Data are presented as count (percentage) or median (interquartile range)

*ARDS* acute respiratory distress syndrome, *CIPNM* critical illness polyneuromyopathy, *GCS* Glasgow Coma Scale, *HIV* human immunodeficiency virus, *ICU* intensive care unit, *SOFA* Sequential Organ Failure Assessment

Patients were awake, following commands, and able to participate in MRC strength testing at only 35% of study visits. A total of 17 patients (18%) were diagnosed with probable CIPNM, and only 1 of these 17 patients could participate in voluntary EMG and MRC testing before discharge to determine definitively if they had neuropathy, myopathy, or both. Of the 17 patients diagnosed with probable CIPNM, 15 were diagnosed at their first study visit (the other 2 were diagnosed at study day 14 and day 21, respectively). Patients who developed probable CIPNM had fewer 28-day ICU-free days (0 (IQR 0–1.5) vs 8 (0–17), *p* = 0.007), were less likely to be discharged home (6% vs 32%, *p* = 0.03), and were more likely to die in the ICU (47% vs 18%, *p* = 0.01) or hospital (53% vs 19%, *p* = 0.004) when compared with patients who did not develop CIPNM. There was no difference in days on mechanical ventilation (10 (IQR 6.5–14.5) vs 9 (6–17), *p* = 0.67). The incidence of probable CIPNM did not differ significantly by admitting ICU (*p* = 0.49), and there was a similar probable CIPNM incidence in the medical (18%) and neurosurgical (21%) ICUs.

There were no statistical differences in the distribution of the right and left CMAP amplitudes for the peroneal nerve (0 mV (IQR −0.6 to 0.4)) and SNAP amplitudes for the sural nerve (0 μV (0–0)), and so the right and left amplitude values were averaged when they were both obtained. Using the previously reported most accurate cutoff amplitudes for the peroneal and sural nerves (0.65 mV for peroneal and 4 μV for sural) [11], the peroneal motor nerve had a sensitivity of 94% (95% CI 71–100%) and specificity of 91% (95% CI 82–96%) for diagnosing probable CIPNM compared with the reference standard, while the sural sensory nerve had a sensitivity of 100% (95% CI 80–100%) and specificity of 42% (95% CI 31–54%). The peroneal motor nerve had a positive predictive value of 70% and negative predictive value of 99%, whereas the sural sensory nerve had a positive predictive value of 27% and negative predictive value of 100% (Table 2; Additional file 1: Figure S1 and Additional file 2: Figure S2). The sensitivities were unchanged when using cutoff amplitudes of 80% of the lower limit of normal for our laboratory (Table 3). The global accuracy of each test (the sum of true positives and true negatives divided by the total population) was 92% for the peroneal motor nerve and 53% for the sural sensory nerve.

**Table 2:** Results of index tests and reference standard

Peroneal motor < 0.65 mV	Sural sensory < 4 μV	No CIPNM	Probable CIPNM
No	No	32	0
No	Yes	39	1
Yes	No	1	0
Yes	Yes	6	16

*CIPNM* critical illness polyneuromyopathy

**Table 3:** Sensitivity and specificity of each nerve amplitude for the diagnosis of probable CIPNM

Nerve	Cutoff amplitude	Normal amplitude	Sensitivity (95% CI)	Specificity (95% CI)
Peroneal motor	0.65 mV	1 mV	94% (71–100%)	91% (82–96%)
Peroneal motor	0.8 mV	1 mV	94% (71–100%)	90% (81–95%)
Sural sensory	4 μV	10 μV	100% (80–100%)	42% (31–54%)
Sural sensory	8 μV	10 μV	100% (80–100%)	31% (21–42%)

*CI* confidence interval, *CIPNM* critical illness polyneuromyopathy

The 67 patients in the medical, cardiac, and surgical ICUs who underwent muscle ultrasound had a median of one study performed per patient; 33 patients (49%) had the worst echogenicity score in any muscle of 2, and no muscles had scores of 3 or 4. A muscle ultrasound echogenicity score in any muscle of at least 2 out of 4 had a sensitivity of 82% (95% CI 48–98%) and specificity of 57% (95% CI 43–70%) for diagnosing probable CIPNM compared with the reference standard, along with a positive predictive value of 27% and negative predictive value of 94% (Additional file 3: Figure S3). The global accuracy of the test was 61%. In all of the patients with probable CIPNM and abnormal echogenicity, the sural and peroneal single NCS were both abnormal. Increased echogenicity was associated with a reduced likelihood of discharge to home (9% vs 50%, *p* = 0.0001) and fewer ICU-free days (3 (IQR 0–15) vs 16 (9.3–19.3), *p* = 0.0002) along with increased ICU mortality (42% vs 12%, *p* = 0.004). We then determined if ultrasound added prognostic information to that obtained from NCS/EMG. For the main outcome of hospital discharge disposition, abnormal muscle ultrasound echogenicity was associated with a lower chance of discharge to home (odds ratio 0.42 (95% CI 0.2–0.86), *p* = 0.02) after adjustment for all the other predictors in the multivariable regression model. For the secondary outcomes, there were no significant associations between abnormal muscle ultrasound echogenicity and ICU-free days or ICU mortality. Only 13 patients had repeated studies of muscle thickness, so we could not examine changes in muscle thickness in this cohort.

The only adverse event was one thigh hematoma after EMG that did not expand after holding pressure and did not require further treatment intervention.

## Discussion

In this prospective observational cohort study, we enrolled 95 heterogeneous critically ill intubated patients and performed serial NCS, needle EMG, and muscle ultrasound to examine simplified screening tests for probable CIPNM. Individual peroneal CMAP and sural SNAP amplitudes had good sensitivity for identifying patients with probable CIPNM, and abnormal muscle echogenicity was a good screening test for probable CIPNM and a predictor of prognosis.

CIPNM is associated with a number of adverse patient outcomes, including prolonged time on mechanical ventilation, longer ICU and hospital stays, increased hospital mortality, higher hospital costs, and a lower likelihood of discharge to home [6, 7, 10, 11, 15, 16, 39–42]. Following hospital discharge, CIPNM is also associated with increased 1-year mortality [17, 41]. Furthermore, in survivors of acute respiratory distress syndrome (ARDS) examined 5 years after their initial illness, physical function was diminished whereas pulmonary function largely returned to normal [43].

Our group previously published a prospective observational cohort study of medical ICU patients with severe sepsis and/or acute respiratory failure requiring mechanical ventilation to determine which specific motor or sensory nerves accurately screened for CIPNM [11]. Using an amplitude cutoff value from receiver operating characteristic (ROC) curves of 0.65 mV for the peroneal CMAP and 4 μV for the sural SNAP, the unilateral peroneal motor nerve was 94% sensitive and 74% specific and the sural sensory nerve was 94% sensitive and 70% specific for diagnosing CIPNM. Latronico et al. also explored the use of unilateral peroneal motor NCS as a screening test for CIPNM in a diverse ICU population and validated the results in a primarily neurological ICU population. This group also found the peroneal nerve to have excellent sensitivity (100%) for CIPNM diagnosis with good specificity (67–85%) [12, 13]. Our study validates those prior results and demonstrates their generalizability to a broader critically ill patient population including patients with sepsis, neurologic emergency, and postoperative respiratory failure. As one of the longest nerves in the body, the utility of the peroneal nerve for diagnosing CIPNM may be partially explained by its vulnerability to tissue ischemia [13].

Critically ill patients experience both muscle wasting and a change in muscle appearance on ultrasound (increased echogenicity) [20–24]. Measurements of muscle ultrasound thickness and echogenicity have high inter-rater reliability in both healthy [25–27] and critically ill patients [28, 29]. Even in the presence of critical illness and edema, muscle thickness measurements at the biceps, mid-forearm, and mid-thigh correlate well with lean body mass [27, 30]. Muscle thickness decreases faster in critically ill patients with multi-organ failure [31]. Increased muscle echogenicity may be caused by intramuscular inflammation, necrosis, edema, fatty deposition, and/or fibrosis [29, 32, 33]. Our study demonstrates that increased echogenicity is a good screening test for probable CIPNM and is associated with deleterious outcomes.

Strengths of our study include generalizability through enrollment of a broad population of critically ill patients with a variety of diagnoses from multiple ICUs. We excluded patients with known pre-existing neuromyopathy through medical record searches and questioning of patients and proxies, supporting the validity of our findings. Single nerve screening tests for CIPNM should only be used in patients without pre-existing neuromuscular disease.

Our study has a number of limitations. The physician performing the electrophysiologic tests was unblinded, although muscle ultrasound was always performed before NCS/EMG at each weekly visit so that electrophysiologic testing results would not influence ultrasound interpretation. Since peroneal and sural NCS results were included as part of the reference standard criteria for probable CIPNM and both the index and reference tests were performed by the same specialist, the study was at risk of incorporation bias, which may lead to overestimation of diagnostic accuracy of our screening tests. We did not perform nerve or muscle biopsies, but it is impractical to perform these invasive procedures in most critically ill patients. It is possible that some of the changes in muscle echogenicity were due to edema, but fluid overload itself may still be harmful [44]. The prevalence of probable CIPNM in our study was 18%, which was slightly lower than the 20% prevalence we expected in our sample size calculations and led to wider confidence intervals for index test sensitivity. We could not perform MRC strength testing due to altered mental status in the majority of study visits, consistent with prior literature demonstrating that most prolonged mechanically ventilated patients are unable to perform manual muscle testing [18]. The inability to perform manual muscle testing gives NCS and muscle ultrasound a potential advantage, as these tests require no active patient cooperation.

There are a number of implications of our study for clinical management. Compared with full four-limb NCS/EMG, unilateral single nerve NCS is quicker, less painful for the patient, and could facilitate the diagnosis of CIPNM. The peroneal CMAP amplitude is 100- to 1000-times larger than the sural SNAP amplitude and is thus easier to find in ICUs that frequently have electrical interference. However, an abnormal peroneal or sural NCS requires follow-up with full NCS/EMG (and ideally muscle strength testing) to confirm a CIPNM diagnosis. Abnormal muscle echogenicity is a good screening test for probable CIPNM and provides additional prognostic information to NCS/EMG. Simplifying the diagnosis of CIPNM with single nerve NCS or muscle ultrasound would have a dramatic impact on clinical practice, leading to earlier diagnosis and increased recognition of CIPNM, better prognostication for patients and families, and more targeted use of treatments such as physical therapy.

## Conclusions

Peroneal motor and sural sensory single NCS are accurate diagnostic tests for probable CIPNM, and muscle ultrasound echogenicity adds value for outcome prediction. Future studies should examine whether these simplified tests can identify good candidates for early ICU physical therapy or be used to monitor therapeutic response.

## Additional files

Additional file 1: **Figure S1.** STARD flow diagram for 95 patients undergoing sural NCS index test. (DOC 43 kb)

Additional file 2: **Figure S2.** STARD flow diagram for 95 patients undergoing peroneal NCS index test. (DOC 43 kb)

Additional file 3: **Figure S3.** STARD flow diagram for 67 patients undergoing muscle ultrasound echogenicity index test. (DOC 44 kb)

## Abbreviations

ARDS: Acute respiratory distress syndrome
CI: Confidence interval
CIPNM: Critical illness polyneuromyopathy
CMAP: Compound motor action potential
EMG: Electromyography
GCS: Glasgow Coma Scale
ICU: Intensive care unit
ICUAW: Intensive care unit-acquired weakness
IQR: Interquartile range
MRC: Medical Research Council
NCS: Nerve conduction studies
ROC: Receiver operating characteristic
SNAP: Sensory nerve action potential
SOFA: Sequential Organ Failure Assessment
