# Supplementary material for: What is the diagnostic accuracy of single nerve conduction studies and muscle ultrasound to identify critical illness polyneuromyopathy: a prospective cohort study
Source: Crit Care. 2018 Dec 17;22:342. doi: 10.1186/s13054-018-2281-9 (PMC6296115; doi:10.1186/s13054-018-2281-9)
Supplement: Supplementary file 2 — Figure S2. STARD flow diagram for 95 patients undergoing peroneal NCS index test. (DOC 43 kb) [file 13054_2018_2281_MOESM2_ESM.doc]

Met inclusion criteria (n=255)

Excluded (n=155)

♦  Declined to participate or could not obtain informed consent (n=98)

♦  Pre-existing neuromyopathy (n=29)

♦  Pharmacologic paralysis (n=4)

♦  Other reasons (n=24)

Enrolled (n=100)

Excluded (n=5)

♦  Found to have pre-existing neuromyopathy (n=5)

No index test (n=0)

Included (n=95)

Peroneal NCS index test (n=95)

Index text positive

(n=23)

Index text negative

(n=72)

Index text inconclusive

(n=0)

No reference

standard (n=0)

Reference standard of probable CIPNM (n=72)

Final diagnosis

-CIPNM present (n=1)

-CIPNM absent (n=71)

-Inconclusive (n=0)

No reference

standard (n=0)

Reference standard of probable CIPNM (n=23)

Final diagnosis

-CIPNM present (n=16)

-CIPNM absent (n=7)

-Inconclusive (n=0)

No reference

standard (n=0)

Reference standard of probable CIPNM (n=0)

Final diagnosis

-CIPNM present (n=0)

-CIPNM absent (n=0)

-Inconclusive (n=0)
